# Biological Control and Plant Growth Promotion Properties of Volatile Organic Compound-Producing Antagonistic *Trichoderma* spp.

**DOI:** 10.3389/fpls.2022.897668

**Published:** 2022-07-26

**Authors:** Jin Ho Joo, Khalid Abdallah Hussein

**Affiliations:** ^1^Soil Biochemistry Lab, Department of Biological Environment, Kangwon National University, Chuncheon, South Korea; ^2^Botany and Microbiology Department, Faculty of Science, Assiut University, Asyut, Egypt

**Keywords:** volatile organic compounds, plant growth-promotion, fungi, environment, agriculture

## Abstract

*Trichoderma* is environmentally vital due to their plant growth-promoting effects (such as enhancement of nutrients supply, suppression of plant pathogens, and promotion of plant defense). Biogenic volatile organic compounds (VOCs) are diverse chemical substances emitted by *Trichoderma* spp. The potential role of VOCs in biological control and plant growth promotion has recently been recognized. Here, the *Trichoderma*-VOCs' performance for plant growth promotion and suppression of plant pathogens are evaluated. We further investigated VOC emission profiles of *T. harzianum* using GC–MS. The *Trichoderma*-VOCs exhibited significant (*p* < 0.05) antifungal properties against all tested pathogenic fungi. *T. atroviride*-VOCs showed a decisive inhibition of *Alternaria panax, Botrytis cinerea, Cylindrocarpon destructans*, and *Sclerotinia nivalis*. The germinating seeds demonstrated growth enhancement in the presence of *Trichoderma*-VOCs emitted by different strains. Low levels of cyclopentasiloxane, decamethyl, cyclotetrasiloxane, and octamethyl were found in *T. harzianum* KNU1 strain whereas cyclopentasiloxane, decamethyl, cyclotetrasiloxane, and octamethyl showed higher emission levels as Si-containing compounds. The results reveal the potentiality of VOCs as a biocontrol resource against deleterious rhizosphere microorganisms and underline the importance of *Trichoderma*-VOCs emissions in regulating plant growth and development.

## Introduction

The reduction of fertilizers and fungicides used in the agricultural field is an insistent demand to maintain the ecosystem and improve sustainable agriculture. Currently, beneficial microorganisms are progressively being used as inoculants for biofertilization and biocontrol. Seed or seedling treatment with both biofertilizers and biocontrol systems has been used to improve the growth of plant crops and minimize the effects on the environment (Kumar et al., 2022). *Trichoderma* is a filamentous fungus that is one of the most widely studied genera with many applications in agriculture and the environment (Mukherjee et al., 2013; Hussein et al., 2020). Several *Trichoderma* species possess the ability to suppress plant diseases along with improving plant growth and productivity *via* overlapping modes of action such as enhanced nutrient efficiency (Zin and Badaluddin, 2020), induced systemic resistance (Farag et al., 2013; Tahir et al., 2017), and mycoparasitism (Mukherjee et al., 2022). In agriculture, *Trichoderma* species are potent biocontrol agents and are often applied to soils to control soil-borne pathogens and increase crop yields worldwide. More than 250 *Trichoderma*-based formulations were estimated in India (Mendoza-Mendoza et al., 2015). Moreover, *Trichoderma* species possess a natural resistance to many chemicals and fungicides used in agriculture, therefore, they are readily integrated into agricultural practices (Liu and Zhang, 2015; Ons et al., 2020). *Trichoderma* species are fertile producers of many metabolites with antibacterial, antifungal and anticancer properties (Khan et al., 2020). *Trichoderma* spp. release a wide spectrum of volatile organic compounds (VOCs). It has been revealed that *Trichoderma* VOCs play a vital role in inter-species communication and can protect plants against some soil-borne pathogens. Although the signaling network between microbes and plants has been extensively studied in the recent two decades, the role of rhizomicrobial VOCs in regulating and enhancing plant growth needs more investigations. Yamagiwa et al. (2011) first reported about the role of VOCs produced by plant growth-promoting fungi (PGPF) in growth promotion. They introduced *Talaromyces wortmannii* as a new PGPF, having growth promotion traits on several plants, such as *Arabidopsis thaliana, Brassica campestris*, and *Phaseolus vulgaries*. Microbial volatiles play a proper role in the agricultural environment by promoting plant growth and inducing the resistance mechanism against plant pathogens, without any harmful effect on the environment (Schulz-Bohm et al., 2017; Tilocca et al., 2020). Recently, the role of volatile materials from rhizobacteria in regulating plant growth and development has been widely considered. Ryu et al. (2003) noticed that a blend of VOCs released from specific plant growth-promoting rhizobacteria (PGPR) strains promoted the growth of the seedlings of *A. thaliana*. Gutiérrez-Luna et al. (2010) also mentioned that airborne chemicals from some *Bacillius* sp. strains have a growth enhancement effect. Farag et al. (2013) found that bacterial volatile compounds are involved in plant growth promotion during their assessment *in vitro* for rhizobacteria. *Trichoderma* spp. are known as PGPF having the ability to promote plant health and to compete against pathogenic microbes (Bissett et al., 2015; El-Maraghy et al., 2020a). The genus *Trichoderma* comprises numerous species (Bitas et al., 2013) which are ubiquitously existent in agricultural soils and forests, where they intensively interact with plant roots and rhizosphere microorganisms (Contreras-Cornejo et al., 2016). A profound understanding of the *Trichoderma* properties, including metabolic activity and the interaction with plants and other microbes, can guarantee its effective use in agriculture (Tyskiewicz et al., 2022). The interest in the application of *Trichoderma* is growing fast due to their direct and indirect biocontrol activity against a wide range of soil-borne disease (Tyskiewicz et al., 2022). Several studies investigated *Trichoderma's* potential to control soil phytopathogens through various complicated mechanisms, such as mycoparasitism, competition for nutrients and space, the degradation of pathogen cell walls, and induction of plant resistance, VOCs also seem a promising approach. Moreover, most studies have been interested in VOCs released from PGPR and their impact on the plant-pathogens relationship. Little is known about plant growth promotion and resistance offered by VOCs-producing PGPF (Tilocca et al., 2020). In this context, this study aimed to detect the plant growth promotion and the biological control properties of the airborne chemicals released from *Trichoderma* rhizosphere strains.

## Materials and Methods

### Sampling and Fungal Cultures

*Trichoderma* strains were isolated from the Korean ginseng (*Panax ginseng*) and pine (*Pinus koraiensis*) soils (rhizosphere) from different localities within Kangwon-do province, Chuncheon, Korea. The soil samples were precisely taken in zip bags and kept at 5°C. The *Trichoderma* strains were isolated and purified on potato dextrose Rose-Bengal agar (PDA amended with 50 μg/ml Rose Bengal) and grown at 27°C (Heydari and Pessarakli, 2010). Among forty-five isolates, only 10 strains were selected based on their antagonism potentiality as bioagents through dual culture technique. *Trichoderma* spp. were identified according to the cultures' features and the direct microscopic examination (Domsch et al., 1980; Moubasher, 1993). The universal primers ITS1: 5′ TCC GTA GGT GAA CCT GCG G 3′ and ITS4: 5′-TCCTCCGCTTATTGATATGC- 3′ were used for fungal amplification (Herlemann et al., 2011). The PCR amplifications were performed in a thermal cycler for 30 cycles at 94°C for 1 min for DNA denaturation, primer annealing for 30 s at 56°C, and primer extension for 1 min at 72°C. The PCR products were sequenced at Macrogen Inc. The partial gene sequences were matched with the full sequences presented in the GenBank database using the BLAST search (NCBI). The phylogenetic tree structure was shaped using the online tool www.phylogeny.fr (PhyML), and the tree visualization was done using TreeDyn. The investigated identified *Trichoderma* strains have been deposited in the Korean agricultural culture collection (KACC) as shown in Table 1. The fungal phytopathogenic isolates *Alternaria panax, Botrytis cinerea, Cylindrocarpon destructans, Fusarium oxysporum, Sclerotinia nivalis*, and *S. sclerotiorum* were obtained from KACC, Jeonju, Korea. The pathogenic fungi were maintained on 2% malt extract agar (MEA) grown at 24°C and stored at 4°C before further investigation.

**Table 1:** *Trichoderma* strains isolated from rhizosphere soils and their identification.

**Strains**	**Max identity (%)**	**Strain of closest identity**	**Identification**	**Accession No**.
T-KNU1	98%	*Trichoderma harzianum* BGP	*Trichoderma harzianum* KNU1	KACC 42249
T-KNU4	98%	*Trichoderma reesei* SU_Solok	*Trichoderma reesei* KNU4	KACC 40557
T-KNU10	98%	*Trichoderma harzianum* BCS8A	*Trichoderma harzianum* KNU10	KACC 40558
T-H22	100%	*Trichoderma harzianum* BGP115	*Trichoderma harzianum* H22	KACC 40779
T-24	100%	*Trichoderma atroviride* IMI 206040	*Trichoderma atroviride* 24	KACC 40776
T-27	100%	*Trichoderma koningii* T-400	*Trichoderma koningii* 27	KACC 40779
T-19	100%	*Trichoderma virens* Gv29-8	*Trichoderma virens* 19	KACC 40780
T-28	100%	*Trichoderma longibrachiatum* H9	*Trichoderma longibrachiatum* 28	KACC 40798
T-P22	99%	*Trichoderma Pleuroticola* GJS	*Trichoderma Pleuroticola* P22	KACC 42583
T-18	99%	*Trichoderma asperellum* IIPR-Ta10	*Trichoderma asperellum* 18	KACC 43821

### Siderophores Biosynthesis Assay

The CAS (Chrome azurol S) liquid assay was conducted according to Schwyn and Neilands (1987) to detect the biosynthesis of the siderophores quantitatively. The solution was amended to pH 6.8 with a 0.1 M Pipes buffer (Prod. No. P1851, Sigma). The fungal cultures were inoculated into liquid deferrated Czapek's medium and incubated at 25°C in a rotary shaker (ICB-S0420, Co., Ltd. China) at 150 rpm till they reached the maximum stationary phase (5 × 10^7^ spores/ml). Then 0.5 ml culture supernatant was added to 0.5 ml CAS solution and 10 μl 5-sulfosalicylic acid 0.2 M (as shuttle solution) and was mixed carefully. The mixtures were settled for 10 min. The color change was determined by absorbance (A_630_) for the vanish of blue color using a UV-VIS spectrophotometer (Agilent Inc., United States). An un-inoculated culture medium was served as blank and the un-inoculated culture medium containing CAS and shuttle solution was served as a reference. The siderophore production was evaluated based on the following Equation (1):


$$\begin{array}{l} \left(A\text{r}-A\text{s}\right)/A\text{r }100=\;\%\;\text{siderophores units} \end{array}$$

where A_s_ is the absorbance rate of the sample, and A_r_ is the absorbance rate of the reference. The experiment was achieved in triplicates and average values were used.

### Phosphate Solubilization Assay

The precipitated Ca_3_(PO_4_)_2_ on Pikovskaya's agar media (g L–^1^; (NH_4_)_2_SO_4_, 0.5 g; Ca_3_(PO_4_)_2_, 5 g; glucose, 10 g; NaCl, 0.2 g; KCl, 0.2 g; MgSO_4_.7H_2_O, 0.1 g; FeSO_4_, 0.002 g; MnSO_4_, 0.002 g; yeast extract, 0.5 g; agar, 15 g) was used for the qualitative detection of the phosphate-solubilizing *Trichoderma* (Pikovskaya, 1948). All *Trichoderma* spp. were grown at 27 °C as surface-culture in 50 ml conical tubes containing 20 ml of Czapek-Dox broth fortified with 0.1% Tween 80. After 1 week, the cultures were vortexed for 1 min and the inocula size was finally adjusted to reach an optical density of 0.3 McFarland Standard (corresponds to ~5 × 10^5^ Cells/ml) using the spectroscopy (UV-VIS, Hitachi U-2900) at 600 nm (McFarland, 1907). The solidified Pikovskaya's agar media was inoculated by *Trichoderma* spp. using a bacteriological isolation loop. The cultures were incubated for 7 days at 27°C and the developed clear zone was detected (Pingale and Virkar, 2013). Phosphate solubilization was detected quantitatively using Pikovskaya's broth (pH was adjusted to 7.0). About 10 ml of Pikovskaya's solution was inoculated using an isolation loop full of *Trichoderma* spore suspension to unify the inoculi size; cultures were kept at 27°C for 7 days (Pikovskaya, 1948). Then the cultures were centrifuged at 16,099 × *g* for 10 min. The supernatant was filtrated using Whatman No. 2 filter to remove the color impurities. In 24 well plates, equal volumes of Barton's reagent and supernatant were added and left for 10 min, The color intensity was assessed using a colorimeter system (Nanodrop Biotech, United States) at wavelength 430 nm, and the soluble phosphorus quantity was detected using a standard curve (Pingale and Virkar, 2013).

### Indole-3-Acetic Acid Assay

A total of ten different *Trichoderma* isolates were quantitatively tested to biosynthesis IAA according to Brick et al. (1991). *Trichoderma* spp. were grown in Czapek-Dox broth amended with tryptophan (1000 μg ml^−1^), as a nitrogen source, instead of NaNO_3_. In a conical tube (50 ml), 10 ml of the liquid bioassay media was inoculated by actively growing hyphae, incubated at 27°C, and agitated at 150 rpm. After 7 days, each *Trichoderma* culture was centrifuged at 16,099 × *g* for 15 min. Salkowski's reagent was prepared (10 mM FeCl_3_ in 35% HClO_4_) to detect indole derivatives. About 1 ml of the supernatant was added to the same volume of the reagent and kept for 30 min in dark conditions. The optical density was detected using UV spectroscopy (Hitachi U-2900) at 530 nm. A standard curve of IAA concentrations was designed to evaluate the corresponding concentration of IAA released by each *Trichoderma* strain in the bioassay media.

### Pathogen-Fungicide Assay

The inhibitory effects of fenhexamid (50%, Indofil) and mancozeb (75%, Indofil) on the linear growth of pathogenic fungi were investigated using the dilution-plate method. The fungicide solution was prepared in sterile H_2_O and added to the autoclaved PDA (~50°C) to reach the final concentration of 20 μg ml^−1^ for each chemical fungicide (Abdoon et al., 2011). The mixture was decanted into Petri dishes (90 × 15 mm) before solidification. Mycelial agar disc (5 mm) was cutoff from the rim of the freshly growing culture of pathogenic fungi and inoculated on the fungicide-amended media surface. The pathogen-fungicide assays were achieved in three replications; all the cultures were incubated at 24°C for 10 days in darkness. The colonies' diameters were measured and the inhibition indices were evaluated in comparison to control (fungicide-free cultures) (Messgo-Moumene et al., 2015).

### Pathogen-Trichoderma VOC-Exposure Bioassay

The *Trichoderma* isolates were inoculated in malt extract agar media (MEA) and incubated at 27°C. One day later, the covers of the Petri dish were exchanged for the bottoms of the 3-day-old PDA cultures of the phytopathogenic fungi. The two halves/cultures of the plate were wrapped together using impermeable parafilm tape and incubated for 10 days at 24°C. The linear growth of the six pathogenic fungi was detected. The bioassay was achieved in triplicates. The controls were inoculated only with the fungal pathogens. All the bioassay procedures were conducted under light-limited conditions to normalize the *Trichoderma* sporulation (Di Lelio et al., 2021). Observations on the antifungal activities of *Trichoderma* VOCs and the inhibition percentage were reported according to the method of Messgo-Moumene et al. (2015) as shown in the following Equation (2):


$$\begin{array}{l} C_{1}-C_{2}/C_{1}\times 100=\;\text{Inhibition percentage (\%)} \end{array}$$

where, C_1_ is the colony surface area of the uninhibited pathogenic fungus in the control, while C_2_ is the colony surface area of the pathogenic fungus in the dual culture.

### Plant-Trichoderma VOC-Exposure Bioassay

Seeds of *Raphanus sativus* L. (radish plant) were surface sterilized by soaking in 70% ethanol for 3 min, rinsed in sterile distilled water (three times), and placed on Petri dishes (I-plates; Atekuto) containing Murashige and Skoog (MS) salt solid medium (pH was adjusted to 5.7) on one side, and a small Petri dish (35 × 10 mm) containing newly growing *Trichoderma* on PDA was put on the other side. A total of five seeds per plate were transferred to the MS medium side (Murashige and Skoog, 2006). Uninoculated PDA in a small Petri dish was set for control. The I plates were sealed with Parafilm tape and incubated randomly in the growth chamber (DASOL; Scientific Co., Ltd., Korea) which was adapted to 25°C and 12:12-h light: dark (LD) conditions.

### Volatile Metabolites Analysis by Headspace GC–MS

*T. harzianum* KNU1 strain was cultured in 10 ml SPME glass headspace vials (Supelco, Bellefonte, St Louis, United States) containing PDA medium and incubated at 27 C for 7 days. The vials were sealed with a duratool-crimp cap fitted with a silicon/Teflon septum that was previously adopted at 100°C for 30 min. Headspace samples taken from sterile PDA served as a negative control. The analysis of *Trichoderma* VOCs was carried out by an Agilent SPME and GC-MS (GEOL and 7890A GC MSD JP/JMS-Q1050GC) equipped with a split-less injection and a capillary DB-WAX GC column (30 × 0.32 mm and 0.5 μ film thickness). The injection port temperature was set at 250°C and the column oven temperature was programmed in the range of 50–250°C (5°C min^−1^), then elevated to 300°C (5°C min^−1^), ending with 5 min isothermal at 300°C. Helium (1 ml min^−1^) was the carrier gas and all mass spectra were detected at 70 eV. The chemical constituents were identified by matching the mass division patterns of the constituents/components to those of the WILEY reference standards data.

### Statistical Analyses

The experimental design was randomized. Each treatment was carried out with at least three replications and compared with the negative control. The data were analyzed by the variance using Duncan's multiple range test (ANOVA) and *p*-value was evaluated. The bars represented the mean values and standard deviation (SD).

## Results and Discussion

Recently, rhizospheric microorganisms got great attention because of their ability to produce strong antimicrobial volatile compounds. Many rhizosphere fungi are known to produce volatile organic compounds, which include terpenes, hydrocarbons, flavonoids, alkaloids, and cyclohexanes. Many of these compounds displayed anti-microbial activities and fuel production (Naik, 2018; Hussein et al., 2020). The endophytic *Phoma* sp. emitted volatile compounds such as caryophyllene, some sesquiterpenoids, alcohols, and naphthalene derivatives, which completely suppressed species belonging to *Sclerotinia, Ceratocystis*, and *Verticillium* (Strobel et al., 2011). The volatile compounds of the endophytic fungus *Colletotrichum truncatum* showed a strong inhibitory effect on *Fusarium sclerotiorum* (Kumar and Kaushik, 2013).

### Trichoderma Plant Growth-Promotion Properties

**Figure 1:**
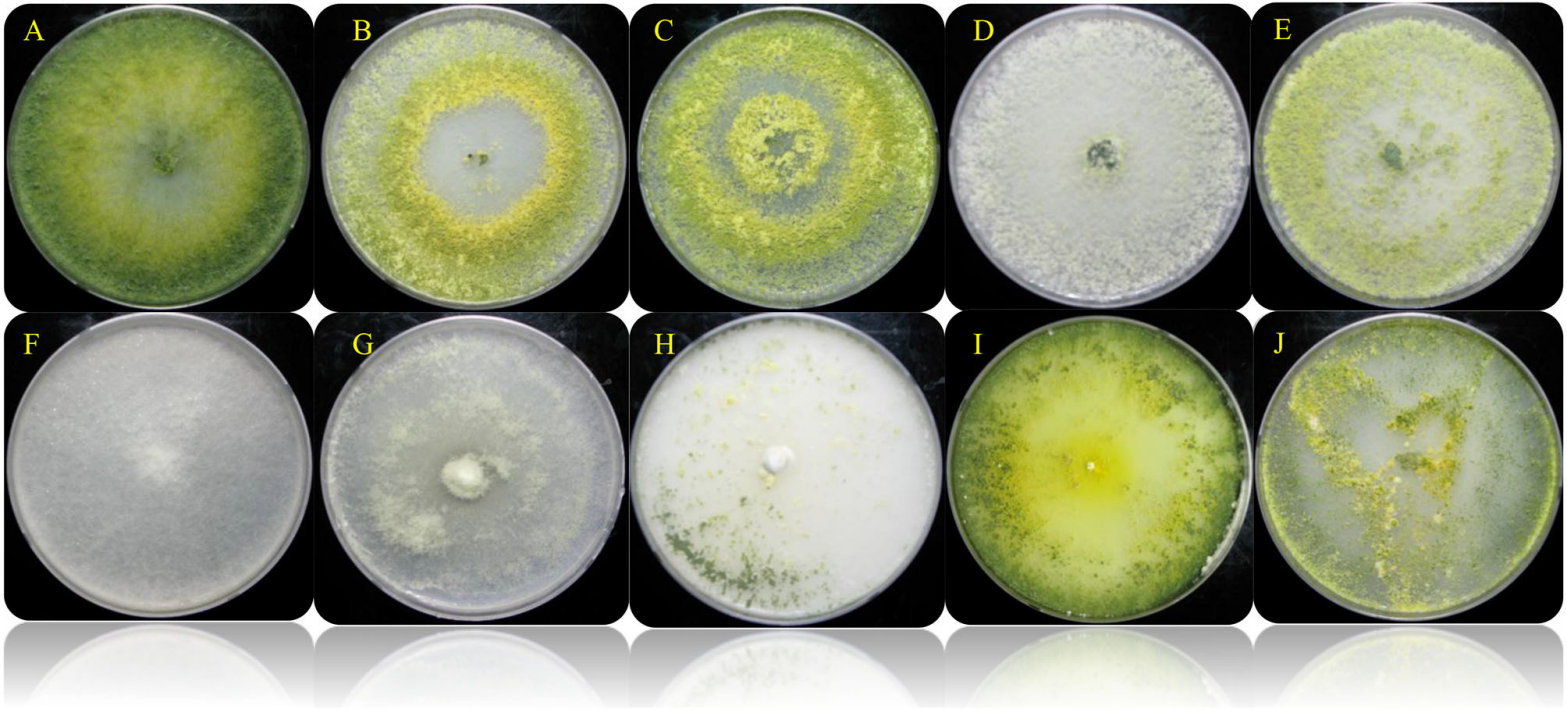
Colony appearance of the different selected *Trichoderma* spp. isolates on PDA (7 days). **(A)** *Trichoderma harzianum* KNU1; **(B)** *T. reesei* KNU4; **(C)** *T. harzianum* KNU10; **(D)** *T. harzianum* H22; **(E)** *T. atroviride* 24; **(F)** *T. koningii* 27; **(G)** *T. virens* 19; **(H)** *T. longibrachiatum* 28; **(I)** *T. Pleuroticola* P22; **(J)** *T. asperellum* 18.

*Trichoderma* is a genus of filamentous fungi, ubiquitous around the world, usually colonizing decaying wood and other forms of plant debris (Howell, 2003). *Trichoderma* is a dominant mycobiome component of various soil ecosystems (such as forests, prairie, farmland, salt marshes, and deserts) in all climatic areas, including temperate and tropical, and the Antarctica regions (Kamala et al., 2015; Ghorbanpour et al., 2018). In this study, ten rhizosphere *Trichoderma* strains (*T. harzianum* KNU1, *T. reesei* KNU4, *T. harzianum* KNU10, *T. harzianum* H22, *T. atroviride* 24, *T. koningii* 27, *T. virens* 19, *T. longibrachiatum* 28, *T. Pleuroticola* P22, and *T. asperellum* 18) that showed dual antagonistic effects and different morphological features were identified and investigated for plant growth-promoting traits, e.g., siderophore production (Figure 1, Table 1). The selected rhizosphere *Trichoderma* strains were varied in terms of their plant growth-promoting traits. For example, the *T. koningii* strain showed the maximum siderophore production (91.7% siderophore units) and solubilized 106.1 μg ml^−1^ of inorganic phosphate. However, *T. asperellum* produced 49.7% siderophore units and solubilized only 2.1 μg ml^−1^ of inorganic phosphate. The lowest siderophore production was by *T. pleuroticola* P22 strain which produced only 20.7% siderophore units (Table 2). The evaluation of the IAA biosynthesis by the selected *Trichoderma* revealed that *T. reesei* KNU4 and *T. harzianum* KNU10 strains produced high amounts of IAA (53.6 and 59.1 μg ml^−1^, respectively) whereas all the other strains produced only <25 μg ml^−1^ of IAA except *T. virens* 19 which produced 36.6 μg ml^−1^. The free living-*Trichoderma* strains isolated from the rhizosphere showed a high capability to mobilize insoluble phosphate. *T. virens* 19 and *T. koningii* strains were potent in phosphate solubilization. They solubilized 152.1 μg ml^−1^ and 106.1 μg ml^−1^, respectively. This result was followed by *T. harzianum* KNU1, which also showed strong phosphate solubilization and liberated 71.1 μg ml^−1^ of inorganic phosphate (Table 2). Generally, fungi synthesize siderophores of hydroxamate-type, such as ferrichromes, coprogens, and fusarinines. *Trichoderma* can inhibit the activity and growth of target soil pathogens by depletion of iron sources in a common niche (Harman et al., 2004). Several representatives of *Trichoderma* are characterized by the capability to synthesize phytohormones and phytoregulators, including indole-3-acetic acid (IAA), which regulates plant development (Ozimek et al., 2018; Jaroszuk-'Sciseł et al., 2019; Alfiky and Weisskopf, 2021).

**Table 2:** Plant growth-promoting activities of the selected rhizosphere *Trichoderma* sp. strains.

**Strains**	**Siderophore**	**IAA production**	**Phosphate solubilization**
	**units (%)**	**μg ml^**−1**^**	**μg ml^**−1**^**
*Trichoderma harzianum* KNU1	29.54 ± 1.95^bc^	21.25 ± 1.72^bc^	71.1 ± 5.11^b^
*Trichoderma reesei* KNU4	31.73 ± 4.31^c^	53.63 ± 15.03^a^	45.02 ± 4.01^bc^
*Trichoderma harzianum* KNU10	33.48 ± 3.18^c^	59.10 ± 6.12^a^	21.52 ± 2.19^c^
*Trichoderma harzianum* H22	40.26 ± 5.11^b^	21.48 ± 15.39^bc^	12.625 ± 1.89^c^
*Trichoderma atroviride* 24	43.11 ± 3.83^b^	18.97 ± 13.63^bc^	12.16 ± 1.14^cd^
*Trichoderma koningii* 27	91.68 ± 5.33^a^	24.60 ± 13.10^bc^	106.17 ± 6.11^a^
*Trichoderma virens* 19	30.2 ± 2.98^c^	36.60 ± 17.04^ab^	152.155 ± 6.89^a^
*Trichoderma longibrachiatum* 28	33.48 ± 2.87^c^	24.51 ± 10.23^bc^	22.485 ± 2.33^c^
*Trichoderma Pleuroticola* P22	20.69 ± 2.21^d^	19.69 ± 3.67^bc^	19.04 ± 2.15^c^
*Trichoderma asperellum* 18	49.67 ± 2.53^b^	10.94 ± 9.35^c^	2.06 ± 1.91^d^

*Same letters within a column are not significantly different at P < 0.05*.

### Bioactivity of VOCs Produced by Trichoderma

**Figure 2:**
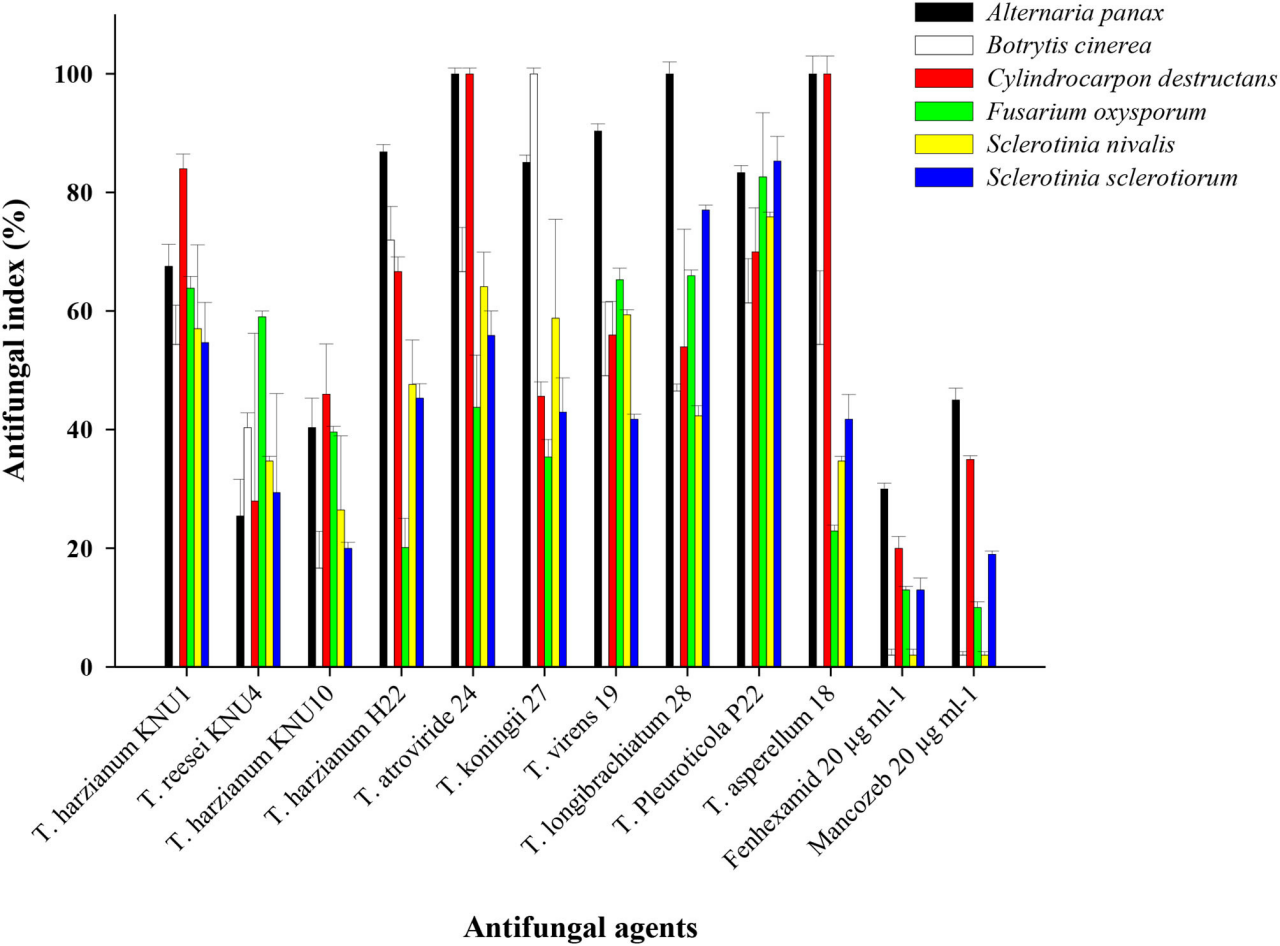
Antifungal activity of the VOCs of *Trichoderma* strains against ginseng-root rot fungi in comparison to fungicides (20 μg ml^−1^) on *B. cinerea, A. panax, C. destructans, F. oxysporum, S. sclerotiorum*, and *S. nivalis*. The antifungal indices were detected after 10 days.

With the constant contact of plants with various phytopathogens, and the increased resistance of these pathogens to chemical pesticides, developing biological protection alternatives became an inevitable demand. Among non-pathogenic microbes, Trichoderma species seem to be the best candidates in the green biotechnology due to their biofertilization and biostimulatory properties. Most *Trichoderma* species belong to plant growth-promoting fungi that can produce phytohormones and the 1-aminocyclopropane-1-carboxylate (ACC) deaminase enzyme (Tyskiewicz et al., 2022). *Trichoderma* can be identified as the genus with the highest biocontrol potential due to many isolated antifungal bioactive compounds and the biostimulation potential of *Trichoderma* (Thambugala et al., 2020; Vishwakarma et al., 2020; Rush et al., 2021). In this study, the pathogen *Trichoderma* VOC-exposure bioassays exhibited significant suppressions to the growth of phytopathogens. The VOCs from *T. Pleuroticola* P22 showed very high inhibition indices against all tested ginseng root-rot fungi. *T. atroviride* and *T. koningii* strains exhibited almost similar plant growth-promoting effects; however, the inhibition (antifungal index) of *T. atroviride*-VOCs appeared to be greater than that of *T. koningii*-VOCs. *T. atroviride*-VOCs and *T. asperellum*-VOCs only exhibited the highest antifungal properties (100% inhibition) against the key phytopathogen of ginseng plant *C. destructans*. The lowest antifungal effect of all *Trichoderma* spp. VOCs was detected in the *T. harzianum* KNU10 strain which showed only 17% inhibition against *B. cinerea* and 20% inhibition against *S. sclerotiorum*. The volatile chemicals emitted by *T. atroviride* exhibited complete inhibition (100%) twice, against *A. panax* and *C. destructans*. Similarly, the *T. asperellum-*VOCs caused 100% growth inhibition against the same phytopathogens. The airborne chemicals released *by T. koningii* and *T. longibrachiatum* showed 100% inhibition of *B. cinerea* and *A. panax*, respectively. The highest antifungal activity against *A. panax* (100% inhibition) was shown by *T. atroviride, T. longibrachiatum*, and *T. asperellum*. The lowest antifungal index was detected in the *B. cinerea* pathogen (16.7% inhibition) by *T. harzianum* KNU10. The volatile compounds blend emitted by *T. harzianum* KNU10 showed the lowest antifungal activity. It inhibited the mycelial growth of *B. cinerea, S. nivalis*, and *S. sclerotiorum* by inhibition percentages of only 16.7, 26.5, and 20%. The chemical fungicide Mancozeb was found to be more effective than fenhexamid, showing 2–45% inhibition indices for all tested phytopathogens. However, fenhexamid showed inhibition indices between 2 and 30% at the same concentrations (Table 3). Generally, the most frequently recommended field concentration of fungicides is between 3 and 35 μg ml^−1^ (Abdoon et al., 2011; Aleksova et al., 2021). The two chemical fungicides used in this study exhibited inhibition indices of <50% in mycelial growth at 20 μg ml^−1^. However, the inhibition index of *Trichoderma*-VOCs on the fungal growth reached 100% several times (Figure 2). For example, both *T. atroviride-*VOCs and *T. asperellum-*VOCs achieved 100% inhibition indices twice against the tested phytopathogens (Table 3). Also, the lowest inhibition percentage of *T. pleuroticola*-VOCs was 61.4% against *B. cinerea*. Consequently, *Trichoderma*-VOCs can exhibit more benefits in the terms of biological control and plant growth promotion over sustainable agriculture.

**Table 3:** The antifungal effect of *Trichoderma* spp. and chemical fungicide against the phytopathogenic fungi.

**Antifungal agents**	***Alternaria***	***Botrytis***	***Cylindrocarpon***	***Fusarium***	***Sclerotinia***	***S. sclerotiorum***
	***panax***	***cinerea***	***destructans***	***oxysporum***	***nivalis***	
*Trichoderma harzianum* KNU1	67.5 ± 3.7^a^	54.4 ± 2.5^a^	84 ± 6.6^a^	63.9 ± 1.96^a^	57.1 ± 14.14^ab^	54.7 ± 6.78^a^
*Trichoderma reesei* KNU4	25.4 ± 6.2^c^	40.4 ± 2.5^ab^	28 ± 28.3^bc^	59.1 ± 0.98^a^	34.7 ± 0.83^c^	29.4 ± 16.64^c^
*Trichoderma harzianum* KNU10	40.4 ± 5^a^	16.7 ± 6^c^	46 ± 8.5^a^	39.6 ± 0.98^b^	26.5 ± 12.48^bc^	20 ± 1^cd^
*Trichoderma harzianum* H22	86.8 ± 1.2^a^	72 ± 5.7^a^	66.7 ± 2.5^a^	20.1 ± 4.91^c^	47.7 ± 7.49^ab^	45.3 ± 2.5^ab^
*Trichoderma atroviride* 24	100 ± 1^a^	66.7 ± 7.4^a^	100 ± 1^a^	43.8 ± 8.84^b^	64.1 ± 5.82^a^	55.9 ± 4.16^a^
*Trichoderma koningii* 27	85.1 ± 1.2^a^	100 ± 1^a^	45.6 ± 2.5^a^	35.4 ± 2.95^b^	58.8 ± 16.64^a^	42.9 ± 5.82^bc^
*Trichoderma virens* 19	90.35 ± 1.2^a^	49.1 ± 12.41^a^	56 ± 5.7^a^	65.3 ± 1.96^a^	59.4 ± 0.83^a^	41.8 ± 0.83^bc^
*Trichoderma longibrachiatum* 28	100 ± 2^a^	46.5 ± 1.24^a^	54 ± 19.8^a^	66 ± 0.98^a^	42.4 ± 1.66^c^	77.06 ± 0.83^a^
*Trichoderma Pleuroticola* P22	83.3 ± 1.2^a^	61.4 ± 7.44^ab^	70 ± 7.4^a^	82.6 ± 10.8^a^	75.9 ± 0.83^a^	85.3 ± 4.16^a^
*Trichoderma asperellum* 18	100 ± 3^a^	54.4 ± 12.41^a^	100 ± 3^a^	22.9 ± 0.98^c^	34.7 ± 0.83^c^	41.8 ± 4.16^b^
Fenhexamid (20 μg ml^−1^)	30 ± 1^d^	2 ± 1^e^	20 ± 2^e^	13 ± 0.58^d^	2 ± 1^e^	13 ± 2^d^
Mancozeb (20 μg ml^−1^)	45 ± 2^c^	2 ± 0.58^e^	35 ± 0.6^c^	10 ± 1^d^	2 ± 0.6^e^	19 ± 0.58^d^

*Same letters are not significantly different at P < 0.05*.

**Figure 3:**
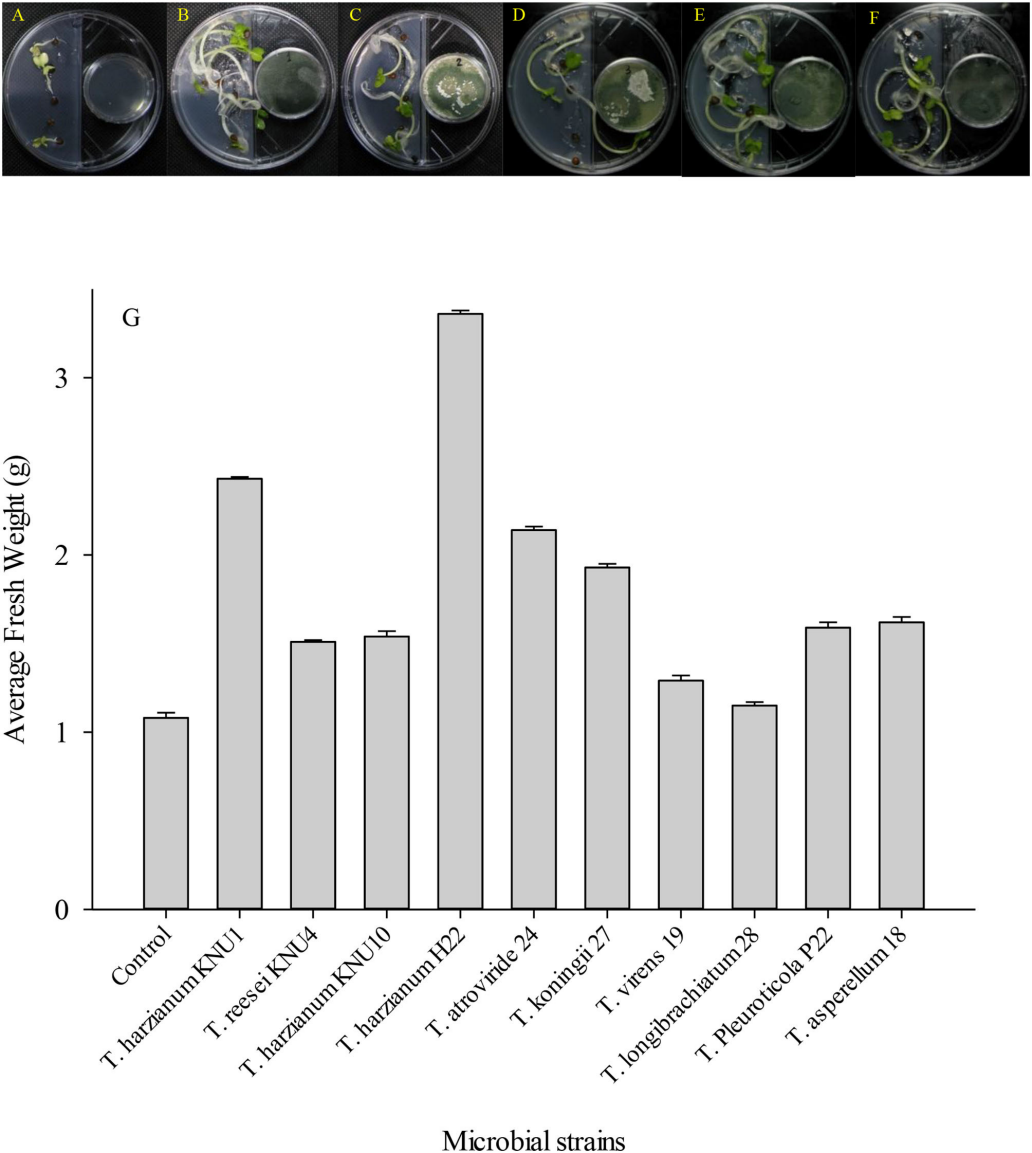
The development of *Raphanus sativus* L. seeds in Murashige and Skoog solid medium in response to plant growth-promoting *Trichoderma*-volatiles exposure of **(A)** Un-inoculated control; **(B)** *T. harzianum* KNU1; **(C)** *T. reesei*; **(D)** *T. harzianum* KNU10; **(E)** *T. harzianum* H22; and **(F)** *T. atroviride*. The I-plates were sealed with Parafilm tape and incubated randomly in the growth chamber which was adapted to 25°C and 12:12-h (LD) cycle. **(G)** Fresh weight average induced in radish seeds by the VOCs released by different *Trichoderma* strains.

**Figure 4:**
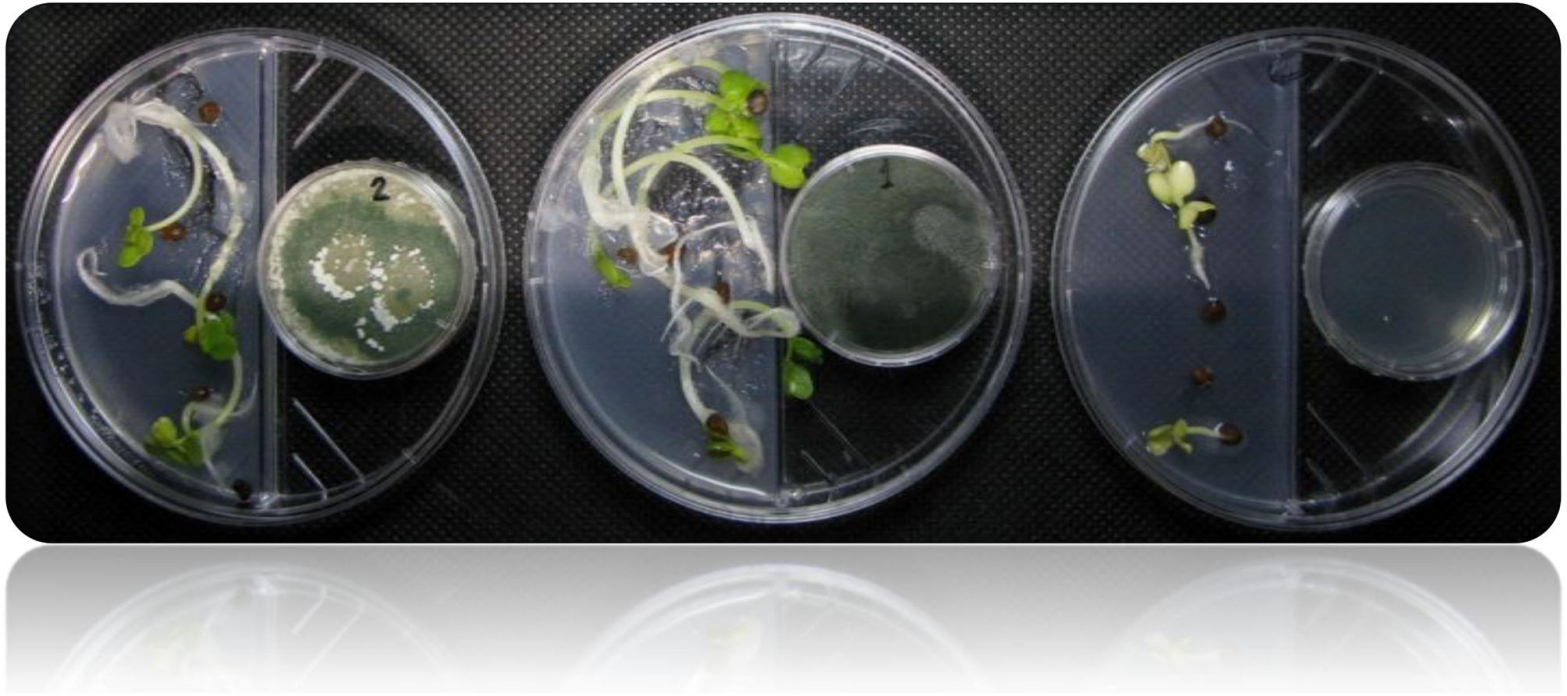
Shows the I-plate system used for assessing plant growth promotion in response to plant growth-promoting *Trichoderma*-volatiles exposure. This setup allows only volatile compounds to be exchanged, while preventing any diffusion of non-volatile metabolites through the medium.

In addition to the composition of the nutrient media, the composition of the gaseous emission is another effective factor for the expedient growth and development of plants (Yamagiwa et al., 2011). Different volatile compounds are present in this gaseous emission, such as nitrogen, carbon dioxide, oxygen, and other types of volatile components produced by surrounding organisms, involving the plant itself (Naznin et al., 2013). In this study*, Trichoderma*-VOCs significantly enhanced the early germination of *R. sativus* seeds. Plant-*Trichoderma* and pathogens-*Trichoderma* VOC-exposure bioassays revealed (39.8–210%) increase in plant biomass and (16.7–100%) suppression of phytopathogens, respectively. The VOCs emitted by *T. harzianum* H22 were associated with the greatest *R. sativus* growth promotion. It increased the fresh weight of the early germinated seeds by more than 200%. However, the volatile compounds emitted by *T. reesei, T. virens*, and *T. longibrachiatum* showed relatively low seedling development. They increased the fresh weight of early germinated seeds by only 39.8%, 19.4%, and 6.5%. In this investigation, *T. virens* and *T. longibrachiatum* did not show a significant increase in the fresh weight of *R. sativus* early germinated seeds (Figure 3). However, these two strains showed the highest antifungal index (inhibition indices) against the ginseng root-rot fungus *A. panax*. Although the *T. Pleuroticola* strain showed a relatively low plant growth-promoting effect, it produced high pathogen-inhibiting VOCs. Similarly, the germinating seeds exposed to VOCs from *T. Pleuroticola* P22 showed only a 47% increase in seedlings' fresh weight. However, *T. Pleuroticola* P22 showed very high inhibition indices against all tested pathogenic fungi. The VOCs produced by *T. harzianum* KNU1 significantly enhanced the seedling development and suppressed the radial growth in all ginseng root-rot pathogens (Figure 4). More interestingly, VOCs produced by the *T. longibrachiatum* strain exhibited a low plant growth-promotion effect, however, the *T. longibrachiatum*-VOCs significantly suppressed the soil-borne pathogens *A. panax, F. oxysporum*, and *S. sclerotiorum* (Table 3). In 2003, Ryu et al. (2003) exhibited that a mixture of airborne chemicals released from some bacterial strains of the rhizosphere can promote the growth of *Arabidopsis* seedlings. Lee et al. (2016) found that tomatoes exposed to airborne chemicals from *T. viride* (BBA 70239) exhibited a larger plant size, a significant increase in plant biomass (99%), and the development of roots. They also observed that the plant growth was dependent on the exposure period to the fungal volatile compounds (Lee et al., 2016). The major volatile component detected from *Talaromyces-*VOCs was β-caryophyllene (a terpenoid-like compound) which significantly promoted the growth of the turnip plant (Yamagiwa et al., 2011). Both *T. harzianum* KNU10 *T. reesei* KNU4 strains showed the same increase in fresh weight (42%). However, the pathogens *Trichoderma* bioassay exhibited that a mixture of airborne chemicals released from *T. harzianum* KNU10 strain suppressed the phytopathogens' growth more effectively. The airborne chemicals released *T. koningii* strain which exhibited the most effective inhibition against *B. cinerea* (100% inhibition). This result is followed by *T. harzianum* H22 strain by an inhibition percentage of 70%. Changes in these components because of different physiological activities *in vitro* largely affect the biological functions, e.g., photosynthesis of the plant (Brilli et al., 2019; El-Maraghy et al., 2020b). The filamentous fungi isolated from the wheat plant produce volatile constituents that could promote the growth of various plants and suppress diseases (Lyu et al., 2020; El-Maraghy et al., 2021). Subsequently, the VOCs-producing fungus *Muscodor albus* was described to have the capacity for growth improvement and suppression of soil-borne diseases (Alpha et al., 2015).

### Headspace GC–MS Analysis of the Fungal VOCs

The gas chromatography-mass spectrometry analysis (GC–MS) of VOCs from the *T. harzianum* KNU1 strain identified more than 80 different compounds including ketones, sesquiterpenoids, and Si-containing compounds. The VOCs of *T. harzianum* KNU1 were selected for the GC–MS analysis because *T. harzianum* was the most frequented species during the current investigation. Besides that, this species achieved adequate results in all bioassays. The relative chemical compositions of the main components in *T. harzianum* KNU1-VOCs were gamma-cadinene (19.5%), 2-methyl-6-methylene-1,7-octadiene (7.0%), allo-aromadendren (6.4%), (+)-calarene tetramethyl-beta-gurjunen (6.3%), longifolen (5.7%), and alpha-selinene (4.0%) (Table 4). Plants and microorganisms comprise many volatile natural chemicals. These molecules are chemically varied, representing fatty acids, indoles, terpenes, and molecules from other chemical families (Fincheira and Quiroz, 2018). 2-Pentenoic acid, 2-ethyl-, methyl ester, and khusilic acids were detected from *T. harzianum* KNU1-VOCs at a low level of 0.03 and 0.15%, respectively. Mhlongo et al. (2018) reported that the presence of acids at very low levels in volatiles of the plant growth-promoting rhizomicrobes might reflect the redox state of their cells. 1-Butanol, 2,2-dimethyle, and 2-amino-1-phenyl-1-propanol alcohols were produced at lower levels than those of acids. Volatiles varied in quantity and number according to the incubation period. 3-Methyl-butanol and 2-Methyl-propanol formed the major components of the volatiles of the plant growth-promoting fungus *Phoma* sp. GS8-3 (Naznin et al., 2013). The 2,4-Hexadienal and Benzaldehyde, 2,4-bis (trimethylsiloxy) aldehydes were released at 0.07 and 0.56%. 2-Methyl-6-methylene-1,7-octadiene alkene was detected as a major volatile component in *T. harzianum* KNU1-VOCs. Acetonitrile-d3, 2,3-pentadiene, 2,4-dimethyl-, aromadendren, dibenzocarbazole, and chlorodecahydroquinoline hydrocarbons were released at low levels from the *T. harzianum*-VOCs strain, whereas gamma-cadinene was detected as a major component. Dideuteriohexane 1-pentyl-2,2-d2 acetate was detected as a major ester volatile component. Other esters like chlorophenyl 6-(4-morpholinyl) hexyl ether were released but at lower concentrations. *Nodulisporium* sp. fungus uniquely produces many ketone compounds particularly, 1,8 cineole, 1-butanol, 2-methyl, cyclohexane, propyl, and phenyl ethanol alcohol, which are the main ingredients of biodiesel when cultured on PDA. *Nodulisporium* sp. also produces several alkyl alcohols, some hydrocarbons, and a few terpenoids (Mends et al., 2012). Alpha-selinene was detected as a sesquiterpenoid at a relatively high level (4%) and a retention time of 20 min. Four other sesquiterpenoids, e.g., calamenene and alpha-calacorene (naphthalene derivative) were also produced at lower levels (Table 4). The endophytic fungus *Nodulisporium* sp. produces cyclohexene, 1-methyl-4-(1-methylethenyl) and 1,4-cyclohexadiene, 1-methyl-, 1–4 pentadiene along with terpenoids and some alcohols of interest as biofuels under microaerophilic growth conditions (Mends et al., 2012). The GC–MS analyses of the VOCs produced by the rhizofungi produced terpenes including alkenes, alkanes, organic acids, and derivatives of benzene (Naik, 2018). Esters were the most effective family of inhibitory compounds tested against fungi, for example, 1-butanol 3-methyl-acetate was biologically very effective in reducing the growth of *F. solani, S. sclerotiorum, Pythium ultimum*, and *Rhizoctonia solani* (Santra and Banerjee, 2020). Several Si-containing compounds, e.g., oxime, methoxy-phenyl, were found in *T. harzianum* strain at low levels, whereas cyclopentasiloxane decamethyl and cyclotetrasiloxane octamethyl showed higher emission levels. Etienne and Gefu (2009) studied 14 elements including aluminum (Al), boron (B), calcium (Ca), chlorine (Cl), iron (Fe), copper (Cu), potassium (K), manganese (Mn), magnesium (Mg), sodium (Na), sulfur (S), phosphorus (P), silicon (Si), and zinc (Zn), in two potato cultivars with different fertilization regimes. They found that the concentrations of Al, Fe, Na, and Si were higher near the ends of the tubers and lower at the center of the tuber. The Silicon element appeared to be greater near the end of the tuber and distributed evenly in the rest of the tuber (Etienne and Gefu, 2009). Elements such as Al, Fe, Na, and Si showed a higher concentration at the end and a lower concentration at the center of the tuber (Etienne and Gefu, 2009). The potential role of d-cadinene in *Trichoderma* defense needs more investigation (Guo et al., 2019). Similar to d-cadinene, caryophyllene was also detected only when *T. hamatum* showed induction to lettuce plant growth (Minerdi et al., 2011). Guo et al. (2019) investigated the VOC profiles of three strains of *Trichoderma* species, *T. hamatum, T. harzianum, T. velutinum*, and the common ectomycorrhizal fungus *Laccaria bicolor* using the SBSE-GC-MS technique. They found that cadinene emission was restricted to the *T. harzianum* strain. Over 370 secondary metabolites which belong to different families of chemical compounds with strong antagonistic properties have been found to produce *Trichoderma* species (Błaszczyk et al., 2014; Ghorbanpour et al., 2018). The most important of these secondary compounds are peptaibols and polyketides (Sood et al., 2020). They are characterized by the presence of amino alcohols and acylated N-terminus (Zeilinger et al., 2016; Tamandegani et al., 2020). Peptaibol is produced by the non-ribosomal peptide synthetases (NRPSs) (Sood et al., 2020). The three main gene-encoding NPRSs were identified in the *Trichoderma* genomes (Druzhinina et al., 2011). Several *Trichoderma* species were found to produce metabolic compounds belonging to the group of anthraquinones, pyrones, terpenoids, and epipoly piperazines (Siddiquee, 2014; Zeilinger et al., 2016). The identified terpenoids in *Trichoderma* include tetracyclic diterpenes sesquiterpenes, such as trichothecenes, as well as triterpene viridin (Zeilinger et al., 2016). *T. harzianum, T. koningii*, and *T. viride* species produce the volatile antibiotic 6-phenyl-pyrone, which is responsible for the distinctive coconut smell (Błaszczyk et al., 2014). Importantly, *T. aureoviride, T. harzianum*, and *T. viride* produce anthraquinone pigments, such as chrysophanol, emodin, and pachybasin, which possess strong antagonistic properties against pathogenic fungi (Eslahi et al., 2021). Ultimately, VOCs produced by *Trichoderma* spp. may convey great benefits in the prospective development of eco-friendly biopesticides for plant-pathogens control and proposes the likely use of the natural antimicrobial compounds from fungi.

**Table 4:** GC-MS analysis showing the VOCs profile of PGPF strain *Trichoderma harzianum* KNU1.

**Peak**		**Compound**	**RT**	**Concentration**
**No**.			**(min)**	**(%)**
15	Acids	2-Pentenoic acid, 2-ethyl-, methyl ester, (e)-	11.47	0.03
71		Khusilic acid	23.59	0.15
10	Alcohols	1-Butanol, 2,2-Dimethyle	8.195	0.02
22		2-Amino-1-Phenyl-1-Propanol	16.41	0.08
11	Aldhydes	2,4-Hexadienal	9.45	0.07
29		Benzaldehyde, 2,4-bis(trimethylsiloxy)-	16.95	0.56
8	Alkenes	2,4-Hexadiene, 2,5-dimethyl-	6.82	0.03
12		Heptane, 3-methylene-	10.03	0.03
16		1,3-Cyclohexadiene, 2-methyl-5-(1-methylethyl)-	11.53	0.03
17		1,5-Cyclooctadiene, 1,5-dimethyl-	11.75	0.16
32		2-Methyl-6-methylene-1,7-octadiene	17.17	6.97
36		1H-Cycloprop[e]azulene, 1a,2,3,4,4a,5,6,7b-octahydro-1,1,4,7-tetramethyl	17.45	1.32
38		Bicyclo[7.2.0]Undec-4-Ene, 4,11,11-Trimethyl-8-Methylene	17.54	0.73
43		2,5-Dimethyl-3-vinyl-1,4-hexadiene (Santolina triene)	18.11	1.81
44		Cis-1,3-Pentadiene	18.17	1.40
46		8-Isopropyl-1-Methyl-5-M Ethylene-1,6-Cyclodecadiene	18.45	3.57
5	Amines	2-Hexanamine, 4-methyl-	2.68	0.09
23		Phenylpropropanolamine	16.45	0.11
75		Methylamphetamine	28.77	0.03
24	Esters	1,5-Dideuteriohexane 1-Pentyl-2,2-D2 Acetate	16.51	0.47
62		Hexadecamethyl Octasiloxane	20.73	0.04
74		2-Chlorophenyl 6-(4-Morpholinyl)Hexyl Ether	28.45	0.08
77		Ethyl 2-Methyl-3-Oxo-3-(2-Pyridinyl)Propanoate	29.61	0.01
78		Diethyl 7-Amino-6-Oxo-6h-Benzo [C]Chromene-8,9-Dicarboxylate	30.14	0.04
79		Ethyl 2-Phenyl-4-Phenylthio Methylthiazole-5-Carboxylate	30.17	0.01
4	Hydrocarbons	Acetonitrile-d3	2.63	0.04
6		2,3-Pentadiene, 2,4-dimethyl-	4.72	0.06
30		Tricyclo[4.4.0.02,7]dec-3-ene, 1,3-dimethyl-8-(1-methyleth yl)-, stereoisomer	17.01	0.48
41		(+)-Aromadendren	17.89	1.88
42		(+)-Calarene [(+)-Beta-Gurjunen]	17.99	1.23
45		(+)-Calarene Tetramethyl-Beta-Gurjunen	18.31	6.32
47		Allo-Aromadendren	18.53	0.58
48		Allo-Aromadendren	18.59	6.36
49		Longifolen	18.69	5.70
50		Alpha-Amorphene	18.74	2.98
51		Gamma-Cadinene	18.92	19.47
53		Delta-Cadinene	19.16	0.23
61		Aromadendrene	20.49	0.03
63		Delta-Cadinene	20.86	0.10
64	Hydrocarbons	Tetracosamethyl-cyclododecasiloxane	21.00	0.10
65		Gamma-Cadinene	21.16	0.14
66		Alloaromadendrene	21.58	0.05
67		Chlorodecahydroquinoline	21.87	0.69
37		1,4-Methanophthalazine, 1,4,4a,5,6,7,8,8a-octahydro-9,9-dimethyl-	17.54	0.73
7		Di-tert-butyl peroxide	6.15	0.23
13		1, 2, 7, 8-Dibenzocarbazole	10.11	0.04
1	Inorganic compounds	Carbon Dioxide	2.34	1.24
2		Carbon Dioxide	2.45	0.07
3		Carbon Dioxide	2.49	0.15
33	Ketones	Cyclohexa-2,5-diene-1,4-dione, 2-methyl-5-(4-morpholinyl)-	17.27	0.29
34		2,4(3H,8H)-Pteridinedione, 8-(3,5-Dimethylphenyl)-3-Methyl-	17.33	0.34
35		2-(4'-Chloro)Styrylchromone 2,4(3h,8h)-Pteridinedione, 8-(3,5-Dimethylphenyl)-3-Methyl	17.38	0.45
70		2,5-Di-tert-butylhydroquinone	22.40	0.23
52	Sesquiterpenoids	Calamenene	19.00	1.60
54		Alpha-Muurolene	19.22	0.55
55		Alpha-Calacorene	19.32	0.25
59		Tetrahydrosmilagenin	19.88	0.31
60		Alpha-Selinene	20.33	4.01
9	Si-containing compounds	Cyclotrisiloxane, hexamethyl-	7.55	0.04
14		Cyclotetrasiloxane, Octamethyl-	10.72	2.78
18		Pentasiloxane, 1,1,3,3,5,5,7,7,9,9-Decamethyl-	12.72	0.02
19		Cyclopentasiloxane, decamethyl-	13.22	3.13
20		Cyclohexasiloxane, Dodecamethyl-	15.65	1.08
21		3,7,7-Trimethyl-11-Methylenespiro(5.5)Undec-2-E	16.13	0.03
25		Cyclotrisiloxane, hexamethyl-	16.58	2.99
26		Oxime-, methoxy-phenyl-	16.63	1.85
27		2,2,4,4,6,6,8,8-Octamethyl-1,3,5,7,2,4,6,8-Tetraoxatetrasilocane	16.74	2.65
28		2-Oxo-4-Nitrosomethyl-6-Trifluoro-Methyl-1,2-Dihydropyrimidine	16.88	0.96
31		Cyclopentasiloxane, Decamethyl-	17.08	1.25
39		Cyclohexasiloxane, Dodecamethyl-	17.78	0.93
40		3-Isopropoxy-1,1,1,7,7,7-hexamethyl-3,5,5 tris (trimethylsiloxy)tetra siloxane	17.84	0.68
56		Dodecamethyl cyclohexasiloxane	19.51	0.14
57		Dodecamethyl cyclohexasiloxane	19.56	0.23
58		Dodecamethyl cyclohexasiloxane	19.65	0.11
68		Hexasiloxane, dodecamethyl	22.02	0.29
69		Hexadecamethyloctasiloxane	22.18	0.08
72		Octa decamethyl, Cyclononasiloxane	23.93	0.03
73		Octadecamethyl Cyclononasiloxane	24.45	0.07
76		Hexadecamethyl Octasiloxane	28.91	0.08
80		Decamethyl cyclopentasiloxane	31.43	0.08

## Conclusions

**Figure 5:**
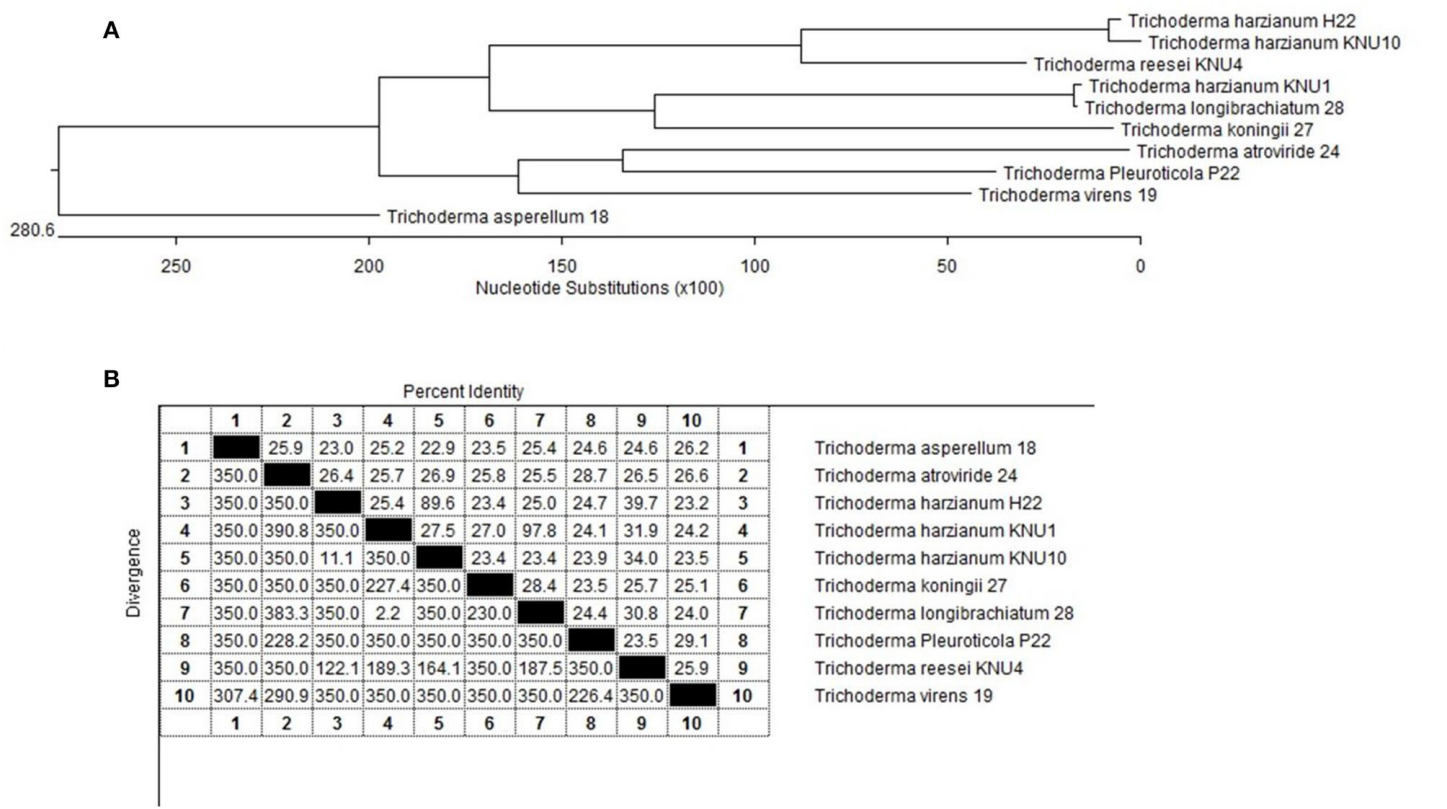
Phylogenetic analysis of the selected *Trichoderma* spp. rhizosphere strains; **(A)** The phylogenic tree showing the lengths of the upright lines are arbitrary; the lengths of the transversal lines are proportional to genetic distances. Bootstrap frequencies are given for a multiple data set of 100 trials; **(B)** 18s rRNA gene sequence similarity for the isolated plant-growth promoting *Trichoderma* strains.

In this study, the role of volatile compounds produced by fungal strains in plant growth promotion and their potential application in biological control were investigated. We identified ten rhizosphere *Trichoderma* strains that showed dual antagonistic effects and different morphological features (Figure 5). Isolated rhizosphere *Trichoderma* strains were investigated for plant growth-promoting traits. The selected rhizosphere *Trichoderma* strains exhibited different capacities in their plant growth-promoting traits. Six plant pathogenic fungi were exposed to volatile organic compounds (VOCs) emitted by *Trichoderma* strains of different species. *T. atroviride*-VOCs and *T. asperellum*-VOCs exhibited complete inhibition against the key phytopathogen *Cylindrocarpon destructans* and *Alternaria panax*. *Raphanus sativus* plant seeds were exposed to volatile organic compounds (VOCs) emitted by the growing culture of *Trichoderma* spp. Exposure to these VOCs emitted by *Trichoderma* strains increased plant biomass by 39.8–210% and suppressed the phytopathogens by 16.7–100%. Low levels of Si-containing compounds, e.g., oxime, methoxy-phenyl, cyclotrisiloxane, hexamethyl, and hexadecamethyl octasiloxane were found in the VOCs of the *T. harzianum* KNU1 strain, whereas cyclopentasiloxane decamethyl and cyclotetrasiloxane octamethyl showed higher emission levels. These results suggest the applicability of *Trichoderma*-VOCs as biocontrol natural products to protect plant crops from soil-borne pathogens and enhance their growth and development.

## Data Availability Statement

The original contributions presented in the study are included in the article/supplementary material, further inquiries can be directed to the corresponding author.

## Author Contributions

Both authors listed have made a substantial, direct, and intellectual contribution to the work and approved it for publication.

## Funding

This work was supported by the National Research Foundation of Korea (NRF) grant funded by the Korean Government (Ministry of Science, ICT, & Future Planning) (NRF-2021R1F1A1063880).

## Conflict of Interest

The authors declare that the research was conducted in the absence of any commercial or financial relationships that could be construed as a potential conflict of interest.

## Publisher's Note

All claims expressed in this article are solely those of the authors and do not necessarily represent those of their affiliated organizations, or those of the publisher, the editors and the reviewers. Any product that may be evaluated in this article, or claim that may be made by its manufacturer, is not guaranteed or endorsed by the publisher.
